# Editorial: Sex differences in aging: a cognitive and behavioral perspective

**DOI:** 10.3389/fnagi.2024.1365482

**Published:** 2024-01-22

**Authors:** Annalena Venneri, Diego Ruano Caballero, Lakshmi Rajagopal

**Affiliations:** ^1^Department of Life Sciences, Brunel University London, Uxbridge, United Kingdom; ^2^Department of Medicine and Surgery, University of Parma, Parma, Italy; ^3^Institute of Biochemistry, Universidad de Sevilla, Seville, Spain; ^4^Pychiatry and Behavioral Sciences, North Western University, Evanston, IL, United States

**Keywords:** Alzheimer's disease, ageing, mild cognitive impairment, MCI, dementia

One among the contributing factors of physiological cognitive decline is aging. Age is the most consistently reported demographic risk factor for Alzheimer's disease (AD) and other forms of neurodegenerative dementia (Guerreiro and Bras, 2015). The effect of aging on cognitive functions is the largest and most consistent influence documented by extensive research (Lipnicki et al., 2013). A strong aging effect is also recognizable when cellular mechanisms are examined (Hou et al., 2019). Research has revealed interesting biological interactions with other genetic, environmental or social factors. An interplay between aging and sex is also frequently reported (Laplume et al., 2022; Alotaibi et al., 2023). There are extensive biological dissimilarities between the sexes as well as differences in cognitive architecture that interact with the aging process influencing differently aging trajectories in males and females. Sex-related differences influence the risk of age-related neurodegenerative conditions that impair cognitive health, with greater risk for AD having been reported in females (Altmann et al., 2014; Mielke, 2018; Burke et al., 2019; Gaugler et al., 2019). Research indicates that females have an increased risk of AD with an odds-risk ratio of 1.56 (95% CI: 1.16–2.10) relative to males (Gao et al., 1998). A possible explanation for this finding might be related to the effects of hormonal changes in older females (Yaffe et al., 2000; Riedel et al., 2016), a hypothesis supported by evidence that hormone replacement therapy leads to a 54% reduction in risk in women in a 16 year longitudinal study (Kawas et al., 1997). Sex differences may also be due to males having an increased risk of vascular impairment (Mccullagh et al., 2001; Peters et al., 2019; Drury et al., 2024) and, therefore, they are less likely to develop a pure form of AD or to differences in longevity between the sexes. Females live longer than males and biological and cognitive divergence of aging trajectories might be influenced by this factor (Hagg and Jylhava, 2021). However, additional evidence suggests that sex-differences in dementia risk might be mediated by other factors such as poor education and greater rates of psychological distress in women (Hasselgren et al., 2020). In contrast, males have twice the risk of developing Parkinson's disease than females, but these latter have faster progression of disease and higher mortality rates (Cerri et al., 2019).

It is important to acknowledge and increase our knowledge of the interaction between aging and sex, given the different sex-related greater vulnerability to neurodegenerative conditions in the aging population. This Research Topic has focused on sex differences in aging from a cognitive and behavioral perspective. It offers a variety of viewpoints leading to a better understanding of the interaction between these biological factors and their differential influence on neurocognitive trajectories. The wealth of evidence emerging from this article Research Topic spans across different perspectives.

Cañete and Giménez-Llort in their transgenic model of AD demonstrate sex-specific early AD-phenotype nociceptive biomarkers. These sex specific biomarkers might explain differences in thermal pain sensitivity in men and women with cognitive decline, providing some theoretical rationale for the decreased pain sensitivity of patients with AD, their increased risk of burn injuries and the observed sex-specific differences in thermal pain discrimination. The authors also suggest that these nociceptive biomarkers might serve as detectors of an early AD-phenotype.

Statsenko et al. attempted to model the trajectories of brain atrophy related to aging in males and females. Their findings suggest sex-specific differences in atrophy, indicating that in males atrophy either starts earlier or has a faster rate than in females. In line with progressing atrophy and consequent enlargement of the interhemispheric fissure, cross-life slowing of decision-making was also observed more in general in all participants. The study also suggests that white matter hyperintensities are not a major determinant of brain structural changes and should be considered as a sign of a disease rather than a common outcome of aging, but sex-specific differences were found, with a stronger correlation and greater number of subcortical and periventricular white matter lesions in women than in men.

Foo et al. detected an overall age related decrease in effectiveness of network communication and loss of functional specialization. They also found that women retained more efficient and less segregated networks than men. Their findings suggest that men may have greater vulnerability to cognitive decline with age. These findings contrast with the extensive evidence of greater risk for cognitive decline in women. However, men and women in the sample included in this study have a relatively high education, supporting the suggestion that the disadvantageous sex-gap in risk for cognitive decline for women might be the outcome of psychosocial disadvantage as suggested by Hasselgren et al. (2020) rather than due to biological factors or greater longevity in women.

Wang et al. looked at whether there was any differential age-related relationship between hypertension accompanied with elevated serum total homocysteine (h-hypertension) and cognition between the sexes. H-hypertension confers a greater risk of cerebral disease. Imaging measures of vascular damage, such as white matter hyperintensities, lacunar infarcts and brain atrophy were greater in females with h-hypertension than in controls but there were no differences in males, suggesting a greater relationship between h- hypertension and subcortical ischemic vascular disease in females.

Santiago et al. took a molecular approach to explore sex-specific differences in switch genes that might contribute to the development of Alzheimer's disease (AD). Studying symptomatic AD patients and individuals with intact cognition but AD neuropathology stratified by sex, these authors found sex-specific transcription factors that might be involved in the pathogenesis of AD. They suggest that there are molecular drivers of sex differences that might account for the sex-specific differences in the development of AD.

The final two articles in this Research Topic take a different approach to clarifying the interplay between aging and sex. Jamalabadi et al. looked at whether parenthood modulated differently the effects of brain aging on aspects of structural brain network controllability in males and females. Brain controllability is a relatively new concept in the field of network neuroscience studying how different neural networks or external factors influence the function of other brain networks involved in cognitive functions. These authors found that parenthood counteracts the effects of aging on brain controllability but the effect is greater in parous women. A unique female characteristic, i.e., giving birth, therefore, may distinctly modulate brain controllability dynamics. Lindseth et al. looked at a number of female-specific factors (e.g., hysterectomy without oophorectomy and use of hormone therapy) and found that all influence aging-related cognitive changes, although effects were mixed and small. APOE was also included in this study and there was a dose dependent effect of the APOE ε4 genotype on aspects of processing speed and executive functioning, but without modifying the relationship between female specific factors and cognitive aging. Stratification by sex was key to the findings in this study.

This article Research Topic offers novel insight, highlights sex differences in cognitive aging and differential vulnerabilities to neurodegeneration between the sexes. The findings stress the need of sex stratification when taking a personalized medicine approach and in research, especially in the field of brain aging.

## Author contributions

AV: Writing – original draft. DR: Writing – review & editing. LR: Writing – review & editing.
